# Supplementary material for: Acute effects of the FIFA11+ and Football+ warm-ups on motor performance. A crossover randomized controlled trial
Source: PLoS One. 2023 Apr 20;18(4):e0284702. doi: 10.1371/journal.pone.0284702 (PMC10118141; doi:10.1371/journal.pone.0284702)
Supplement: S1 Table — (DOCX) [file pone.0284702.s002.docx]

Table 1, Mean values, standard deviation (SD) and mean standard error (mean SE) for the 11+ and Football+

|  |  | Overall | |  |  |  | Females | Males |  | |
| --- | --- | --- | --- | --- | --- | --- | --- | --- | --- | --- |
|  |  | Mean(s) | | SD(s) | Mean SE(s) | Cohen's d | Mean(s) | SD(s) Mean SE Cohens's d Mean(s) SD(s) Mean SE Cohens's d |  | |
| 20 m | 11+ | | 3.26 | 0.23 | 0.04 | 0.42 | 3.39 | 0.21 0.03 3.17 0.19 0.02 |  |  |
|  | Football+ | | 3.17 | 0.44 | 0.07 |  | 3.36 | 0.20 0.22 3.11 0.20 0.61 |  |  |
| IA | 11+ | | 17.68 | 0.95 | 0.15 | 1.43 | 18.31 | 0.82 0.11 17.23 0.77 0.10 |  |  |
|  | Football+ | | 17.03 | 1.03 | 0.17 |  | 17.78 | 0.72 1.24 16.48 0.87 1.62 |  |  |
| DS | 11+ | | 14.33 | 3.24 | 0.52 | 0.38 | 16.29 | 3.25 0.44 12.90 2.42 0.26 |  |  |
|  | Football+ | | 13.72 | 2.53 | 0.41 |  | 14.99 | 3.40 0.73 12.79 2.24 0.09 |  |  |
| CMJ | 11+ | | 31.20 | 7.60 | 1.23 | -0.13 | 28.39 | 6.56 0.95 -0.27 33.98 6.76 0.57 |  |  |
|  | Football+ | | 31.63 | 7.21 | 1.17 |  | 27.36 | 7.16 33.99 6.86 -0.003 |  |  |

SD= standard deviation, SE= standard Error
