# Supplementary material for: Acute effects of the FIFA11+ and Football+ warm-ups on motor performance. A crossover randomized controlled trial
Source: PLoS One. 2023 Apr 20;18(4):e0284702. doi: 10.1371/journal.pone.0284702 (PMC10118141; doi:10.1371/journal.pone.0284702)
Supplement: S1 Appendix — (DOCX) [file pone.0284702.s003.docx]

| *Part one* | *Running exercises 15 min* |
| --- | --- |
| Running forward & backward a cross the pitch | 40-50 percent of maximal pace |
| Side running a cross the pitch | Once each side |
| Dynamic stretches of the hip muscles a cross the pitch | Hip in/out/flexion/abduction/adduction, once each |
| Running shoulder contact | Jump, controlled shoulder contact with partner and landing |
| Controlled lunge across the pitch | Forward and side lunge, once each |
| Dynamic plank exercise (prone position) | 1x 30 |
| Modified NHE | 1x10 (3 submaximal, 7 maximal) |
| Copenhagen exercise | 1x10 each side |
|  |  |
| *Part two* | ***Small sided games 5 min*** |
| Unanticipated dribbling | 2x 30 s at maximal pace |
| Active passing | 2x60 s with an external focus (counting) |
| 1 vs 1 | 1x 30 s at maximal pace |
|  |  |
| *Part three* | ***Anaerobic exercises 5 min*** |
| Plyometric exercises | 180 s |
| Sprinting | 60 s |
| Change of direction | 60 s |

Appendix 1. the Football+ program
