# Supplementary material for: Acute effects of the FIFA11+ and Football+ warm-ups on motor performance. A crossover randomized controlled trial
Source: PLoS One. 2023 Apr 20;18(4):e0284702. doi: 10.1371/journal.pone.0284702 (PMC10118141; doi:10.1371/journal.pone.0284702)
Supplement: Attachment — Submitted filename: Response to reviewers.docx [file pone.0284702.s004.docx]

**Point-to-point Responses to reviewers**

ACADEMIC EDITOR:

Dear authors,
The work is interesting but in the current form, there are several issues that need to be addressed before further consideration. Indeed, there are some important issues that may lead to rejection. Even so, I believe that it is more correct and ethical to give a chance for authors to explain.
Thus, I suggest major revisions.
Please address and answer all comments made by the three reviewers.
Thank you.

**Response to academic editor**

*Dear academic editor,*

*On behalf of my research team, I would like to thank you so much for your positive feedback and considering the work for further assessments. We have tried to address all the points and deficiencies identified by the reviewers and believe that the point you and the reviewers raised would absolutely improve the manuscript quality.*

**Reviewer #1:** Thank you for the opportunity to review this article. The paper addresses a novel under-researched area, which has the potential to provide useful recommendations for coaches. However, there are some questions that need to be addressed to the manuscript.

****dear reviewer 1.*

*On behalf of my research group, I would like to thank you so much for your constructive comments and commentary on our manuscript. We believe that the points you have raised strongly improve the quality of this work. Therefore, our effort was to address all the points and integrate them throughout the manuscript. Revisions made based on your comments are highlighted in yellow throughout the manuscript.*

Specific comments are provided below:

ABSTRACT
**Remove “SPSS V.29”**

****The relevant sentence was revised accordingly.*

**KEYWORDS**
Change the keywords that match in the title (line 26)

****The keywords were changed accordingly*

**INTRODUCTION**
This study focuses on men and women. However, the introduction does not distinguish between the two genders. Please clarify this.
Additionally, please update the references and include the results of meta-analyses related to this topic.

***Thank you so much for your constructive comments. The references were updated, and additional information was added accordingly. Information related to gender was added to the introduction.

**Male or female? (sentence 2, pag 3)**

***males

**Add references (sentence 4, pag 3).**

****The reference was added.*

**Add results of all studies included in the second paragraph**

*** Thank you very much for your comment. The related results were added to the end of the second paragraph.


**METHODS
Participants
Why four participants dropped out during the study period? Add it.**

**** they were excluded due to Coronavirus infection*

**Performance tests
What kind of boots did you use the participants during performance tests?**

****All players put on the soccer shoes that are regularly used in artificial turf*

Include the height of time gates

**** Time gate height was added*

**Statistical analyses
Please divide the sample by gender and perform the statistical analysis.**

****thank you very much for your recommendation.* *We performed the statistical analysis based on gender and reported the outcomes accordingly as per your request. However, the power analysis reveals that the sample needed for this study is 34, reducing the sample based on the gender potentially leads to underpowered results, and consequently, non-significant outcomes came out.*

**The second paragraph of this sections should be in the results section. Change it.**
**** The second paragraph was moved to the result section.*

RESULTS
**Include Cohen's d values in table 1.**

****thank you very much. The values were already mentioned in the text. Meanwhile, they were added to the table as per your request.*

DISCUSSION
**Add a paragraph with the limits of the study and possible future studies.**

***thank you for your constructive comment. A limitation section was added to the manuscript

CONCLUSIONS
**There is not a proper conclusion where the main findings are highlighted. Add guidelines on how relevant are the findings of your study for physical trainer.**

****Thank you so much. The conclusion was revised entirely.*

APPENDIX 1.
**Is it possible to represent each exercise as a figure? That would help the reader to understand it better**

******* Thank you very much for your comment. Unfortunately, the photos for all exercises are not available currently. However, we are planning to make poster for the Football+ program and will publish it once it becomes available.

**Reviewer #2:** First, I would like to congratulate the authors for the pertinence of the study. I will, however, present some suggestions for improvement.

***dear reviewer 2.

On behalf of my research group, I thank you so much for your great feedback and constructive commentary on the manuscript. The points you have raised would absolutely induce the quality of the work, and thus, we tried to integrate them throughout the manuscript. The changes made based on your comments are highlighted in green.

**- Abstract must comply with the rules of the journal**

*** thank you very much. We checked the abstract format.

**- Introduction: Refer what the present study adds to existing knowledge. The objectives must be clarified. According to what exists, is it not possible to establish hypotheses?**

*Thank you very much. We have integrated your suggestion throughout the introduction and revised it accordingly.*

**Methodology: Interventions - I suggest pairing an illustration or table with the programs used.**

****thank you very much. Given that the 11+ is a well-known and somewhat commercial programme and its table and catalog have been published several times in different languages, we assumed that citing the related references would suffice. However, given that two reviewers agreed on this point, we added the 11+ description to the text.*

**Results: Notes should be placed on abbreviations in the table. Improve the table so that all results are clearly displayed.**

****thank you. We added footnotes to the table.*

**Discussion: I suggest that results are not presented in the discussion.**

****thank you. We removed the results from the discussion.*

**Authors should mention the limitations of the study and propose future investigations.**

****thank you. We added a limitation section to the end of the manuscript.*

**Present the practical implications of the study.**

****thank you for your constructive comment. We added a relevant sentence to the end of the discussion*.

**Review the formatting of references.**

****The references were adjusted according to the journal guidelines.*

**Reviewer #3:** Congratulations to the authors. The study has a simple method and findings section. The method section is not written in detail. Given the quality of the journal (my quarterly tranche Q1), I would like to reject the study.

Dear reviewer3.

*On behalf of our research team, I thank you so much for your commentary on this work. The critical points you highlighted increase the quality of the work, and hence, we have tried to address them properly throughout the manuscript. The changes we made based on your comments are highlighted in light blue.*

**- The data collection process cannot be detailed. Where by whom, and at what time of day the data (mornings???) were collected. It should be detailed. How was it applied to a total of 48 people? On different days? If 48 people practiced in the same session, waiting time during different tests may reduce the warming effect. What action was taken for this? The Experimental design should be illustrated?**

****Thank you so much for your constructive comment. Additional information was adjusted to the text as per your suggestions. The measurements were conducted in mornings as the training sessions of the players was also at mornings.*

**-11+ exercises are mentioned in the Interventions section. Like the Football+ programme, it should be presented in detail in the table.**

****thank you very much. Given that the 11+ is a well-known and somewhat commercial programme and its table and catalog have been published several times in different languages, we assumed that citing the related references would suffice. However, given that two reviewers agreed on this point, we added the 11+ description to the text.*

**-What kind of advice was given to the athletes before the measurements (sleep, nutrition). should be explained.**

****thank you for your feedback. We asked the players to not perform heavy physical activities 48 h before measurements, sleep no later than 12 pm the night before measurements and drink only caffeine-free liquids 10 h before measurements. The suggestions were added to the text.*

**Why were men and women evaluated in the same group? Different tables can be created for both genders.**

*** Please divide the sample by gender and perform the statistical analysis.

****thank you very much for your recommendation.* *We have performed statistical analysis based on the gender and reported the outcomes accordingly.*

**In the study, two different protocols were tested on the participants. There could have been 3 different protocols in the study. That is, the study could only have a control group in the form of general warm-up or jogging to determine the effect of other warm-up protocols according to the control group.**

****thank you for your constructive recommendation. This study focused on the acute effects of the 11+ as a gold standard for injury prevention in comparison to a football-specific warm-up, the so-called football+. Given that the previous studies were also compared the 11+ to a dynamic warm up, we assumed that having two structured warm up modalities including different contents might be more practical than having a control group alongside the 11+.*

**How the intensity of the exercise was adjusted during both warm-up protocols in all participants. It should be disclosed.**

***two practitioners who have been doing the 11+ programme for up to 2 years were adjusted the training intensity by asking the players to keep the intensity level as high as the practitioners themselves do. Generally, in this kind of interventions, it is quite common to adjust the intensity based on individual‘s estimations simply because there is no other alternative to control that. The 11+ itself also uses self-estimated approach to control the intensity of the exercises.

**The discussion section is not detailed enough. Physiological mechanisms have not been adequately explained. Up-to-date resources are needed.**

***thank you for your constructive comment. We revised the discussion and integrated additional inputs. References were updated as per your request.
